# Ocular Delivery of Predatory Bacteria with Cryomicroneedles Against Eye Infection

**DOI:** 10.1002/advs.202102327

**Published:** 2021-09-08

**Authors:** Mingyue Cui, Mengjia Zheng, Christian Wiraja, Sharon Wan Ting Chew, Arti Mishra, Venkatesh Mayandi, Rajamani Lakshminarayanan, Chenjie Xu

**Affiliations:** ^1^ Department of Biomedical Engineering City University of Hong Kong 83 Tat Chee Avenue Kowloon Hong Kong SAR China; ^2^ School of Chemical and Biomedical Engineering Nanyang Technological University 62 Nanyang Drive Singapore 637459 Singapore; ^3^ Ocular Infections & Anti‐Microbials Research Group Singapore Eye Research Institute The Academia 20 College Road, Discovery Tower Singapore 169856 Singapore; ^4^ Ophthalmology and Visual Sciences Academic Clinical Program Duke‐NUS Graduate Medical School 8 College Road Singapore 169857 Singapore; ^5^ Department of Pharmacy National University of Singapore 18 Science Drive Singapore 117543 Singapore

**Keywords:** cryomicroneedles, drug delivery, eye infection, ocular disease, predatory bacteria

## Abstract

The development of potent antibiotic alternatives with rapid bactericidal properties is of great importance in addressing the current antibiotic crisis. One representative example is the topical delivery of predatory bacteria to treat ocular bacterial infections. However, there is a lack of suitable methods for the delivery of predatory bacteria into ocular tissue. This work introduces cryomicroneedles (cryoMN) for the ocular delivery of predatory *Bdellovibrio bacteriovorus* (*B. bacteriovorus*) bacteria. The cryoMN patches are prepared by freezing *B. bacteriovorus* containing a cryoprotectant medium in a microneedle template. The viability of *B. bacteriovorus* in cryoMNs remains above 80% as found in long‐term storage studies, and they successfully impede the growth of gram‐negative bacteria in vitro or in a rodent eye infection model. The infection is significantly relieved by nearly six times through 2.5 days of treatment without substantial effects on the cornea thickness and morphology. This approach represents the safe and efficient delivery of new class of antimicrobial armamentarium to otherwise impermeable ocular surface and opens up new avenues for the treatment of ocular surface disorders.

## Graphical abstract

This report introduces a cryo‐delivery platform inside which the predation ability of *Bdellovibrio bacteriovorus* is highly reserved to impede the development of gram‐negative infections.

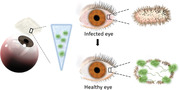

## 1 Introduction

Bacteria are the major etiological agents of ocular infections.^[^ ^1^ ^]^ If left untreated, they can damage the structures of the eye, leading to irreversible visual impairment and blindness. Conventionally, eye infections are treated with antibiotic eye drops. However, the abuse of antibiotics has led to the evolution of antibiotic‐resistant bacteria, casting a shadow on the future of antibiotic‐based treatments.^[^ ^2^ ^]^ One potential solution is to use predatory bacteria that prey on other bacteria. For example, *Bdellovibrio bacteriovorus* (*B. bacteriovorus*), found in the 1960s, was able to reduce the bacterial burden of *Klebsiella pneumoniae* (*K. pneumoniae*) by more than 3.0 log_10_ in the lungs of rats, as measured by colony‐forming unit (CFU) plating.^[^ ^3^ ^]^ Shanks et al. further reported that *B. bacteriovorus* could consume gram‐negative pathogens associated with keratitis (i.e., *Pseudomonas aeruginosa* (*P. aeruginosa*) and *Serratia marcescens*) while being nontoxic, and noninflammatory, in rabbit studies.^[^ ^4^ ^]^ Their preliminary data also demonstrated that the predatory bacteria accelerated the clearance of pathogens from theocular surface.^[^ ^5^ ^]^

Currently, ocular delivery of predatory bacteria is performed through topical instillation.^[^ ^4^ ^]^ Poor penetration, rapid clearance by blinking reflexes and poor patient compliance are the major drawbacks associated with topical instillations. Though topical delivery is suitable for conjunctivitis and keratitis, it is ineffective for the treatment of infections such as endophthalmitis, which requires the migration and deep penetration of the predatory bacteria. Alternatively, intravitreal injection can be performed, especially using microneedles (MNs). These tiny needles allow precise control of the injection depth and area.^[^ ^6^ ^]^ As early as 2009, Prausniz et al. demonstrated this concept by using a glass hollow MN to infuse solutions containing fluorescent molecules or particles into human cadaver sclera in vitro.^[^ ^7^ ^]^ We have reported a polymeric double‐layer MN for both the quick release of anti‐inflammatory drugs and the sustained release of anti‐angiogenic monoclonal antibodies in the cornea.^[^ ^8^ ^]^ Unfortunately, hollow MNs require freshly prepared bacterial formulations, while polymeric MNs are unsuitable for the delivery of living objects.

**Scheme 1:**
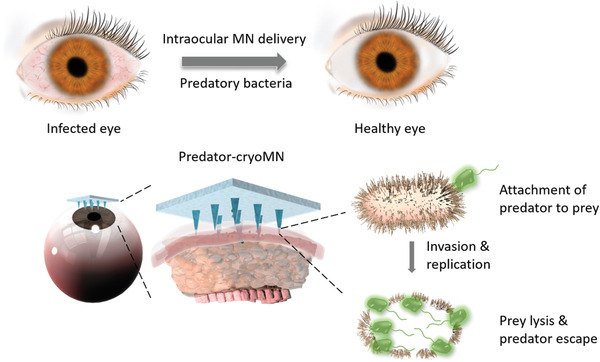
Illustration of cryomicroneedles (cryoMNs) for ocular delivery of predatory bacteria in treating eye infection.

We recently reported a cryomicroneedle (cryoMN) platform technology that successfully delivered living dendritic cells into the dermis layer for cancer immunotherapy.^[^ ^9^ ^]^ In this project, we hypothesize that cryoMNs are also suitable for packing and delivering predatory bacteria for eye infection treatment (**Scheme** **1**). Using *B. bacteriovorus* as the model predatory bacteria, we first optimized the cryoMN formulation to maximize predatory viability while maintaining their mechanical properties for corneal penetration. Later, in vitro experiments were carried out to demonstrate the retention of the predatory ability of *B. bacteriovorus* against gram‐negative bacteria after release from cryoMNs. Four gram‐negative bacteria were studied, namely *Escherichia coli* (*E. coli*), *P. aeruginosa*, *Acinetobacter baumannii* (*A. baumannii*), and *K. pneumoniae*. These bacteria were chosen for their clinical significance. *E. coli* can induce conjunctivitis or dacryocystitis.^[^ ^10^ ^]^ *P. aeruginosa and A. baumannii* are responsible for keratitis resulting from trauma, contact lens wear, or ocular surgery.^[^ ^11^ ^]^ *K. pneumoniae* may spread from the blood and cause endophthalmitis.^[^ ^12^ ^]^ Finally, the therapeutic potential of this formulation was demonstrated in a mouse eye infection model (*E. coli* as the pathogen), using topically applied *B. bacteriovorus* as the control. The infection was significantly decreased by nearly six times through 2.5 days of treatment without affecting the cornea thickness and morphology.

## 2 Experimental Section

Lysogeny broth (LB) agar, agarose, glycerol, calcium chloride, magnesium chloride, polystyrene (PS), polycaprolactone (PCL), polylactic acid (PLA), and paraformaldehyde were obtained from Sigma‐Aldrich (Singapore). Mini hyaluronic acid (miniHA) powder was purchased from Bloomage Freda Biopharm Co., Ltd. (China). Phosphate‐buffered saline (PBS) and 4‐(2‐hydroxyethyl)‐1‐piperazineethanesulfonic acid (HEPES) were purchased from GE Hyclone (Singapore). FM 4–64FX dye was purchased from Thermo Fisher Scientific (Singapore). Nutrient broth and LB were purchased from BD Diagnostics (USA). *B. bacteriovorus* (ATCC 15143), *E. coli* (ATCC 25922), *E. coli* (ATCC 10536, only for *B. bacteriovorus* culturing), *P. aeruginosa* (PAO1‐GFP)*, A. baumannii* (ATCC 19606), and *K. pneumoniae* (ATCC BAA‐2784) were purchased from ATCC (USA).

### 2.1 Culturing of *B. bacteriovorus*

*E. coli* (ATCC 10536) was grown in LB broth with aeration at 37 °C and harvested during the stationary growth phase. *B. bacteriovorus* was grown and maintained using *E. coli* as prey. They were maintained as plaques in double‐layered diluted nutrient broth, a 1:10 dilution of nutrient broth supplemented with 2 × 10^−3^ m CaCl_2_ and 3 × 10^−3^ m MgCl_2_, and agar (0.6% agar in the top layer and 1% agar in the bottom layer, pH = 7.2). Lysates were initiated by co‐culturing a plug of top agar containing *B. bacteriovorus* with washed prey/host cells (*E. coli* ATCC 10536) in HEPES buffer. They were incubated at 30 °C on a rotary shaker until the culture cleared (stock lysates). To obtain higher predator concentrations, fresh predator cultures were obtained as previously described.^[^ ^4^, ^12^, ^13^ ^]^ Briefly, 2 mL of washed overnight culture prey cells (≈1 × 10^9^ CFU mL^−1^) were incubated with 2 mL of stock lysates in 20 mL of HEPES. The co‐cultures were incubated for 24 h before passing three times through a sterilized 0.45 mm Minisart syringe filter (Sartorius) to remove any remaining prey cells and debris to purify the predators. Next, centrifugation was conducted three times at 15 000 rpm for 30 min to concentrate the predator cells. For the last wash, the pellet was re‐suspended in 2 mL PBS solution to reach a final absorbance of ≈0.3–0.4 at 600 nm. The final concentration was determined using the double‐layered agar method each time. Fifty microliter aliquots of the predator samples were plated on LB agar and cultured at 37 °C to confirm the thorough removal of prey cells.

### 2.2 Fabrication of cryoMN Patches with Bacteria

CryoMNs were fabricated using template molding. Briefly, a buffer solution containing predatory bacteria was cast into a PDMS negative mold made from a designed stainless‐steel MN template. The buffer solutions for preparing cryoMNs were composed of PBS, glycerol, and predatory bacteria. The concentration of glycerol ranged from 0% to 20%. The concentration of bacteria ranged from 10^8^ to 10^9^ PFU mL^−1^. Fifty microliters of the optimized formulation containing *B. bacteriovorus* was added to the PDMS negative mold and centrifuged at 4000 rpm for 1 min, driving the solution into the tip cavities. A 20 µL quantity of solution was then added as the base for a 3 × 3 MN mold. The whole system was cooled at 4 °C for 30 min to allow the sedimentation of bacteria from the base, concentrating the bacteria in the MN tips. They were then stored at −20 °C for 4 h prior to prolonged storage at −80 °C. The cryoMN patches could be peeled off from the molds after 4 h of storage at −80 °C.

### 2.3 Mechanical Test

The mechanical strength of the cryoMN patch was evaluated by compression testing using an Instron 5543 Tensile Meter. The MN patch was placed on a flat stainless‐steel platen with tips facing upward. Subsequently, vertical force was applied to the tips at a constant speed of 0.5 mm min^−1^. The displacement versus loading force curve was recorded until a preset maximum force of 4 N per needle was achieved.

### 2.4 In Vitro Predation Experiment

The predatory ability of *B. bacteriovorus* was examined by co‐culturing with gram‐negative bacteria (*E. coli* (ATCC 25922)*, P. aeruginosa* (PAO1‐GFP)*, A. baumannii*, and *K. pneumoniae*) in vitro.^[^ ^4^, ^13^ ^]^ Briefly, co‐cultures were prepared by adding 0.1 mL of HEPES washed prey cells (≈1 × 10^8^ CFU mL^−1^) to 0.1 mL of harvested predators to compare their susceptibility to predation. The cultures were incubated at 30 °C for 48 h. Optical density at 600 nm was recorded throughout the co‐culture process using a BioTek plate reader. Prey ability was evaluated by the reduction in the number of prey cells after co‐culture. Cell viability was quantified by CFU enumeration following dilution plating at 0, 24, and 48 h. Each experiment was conducted three times, with three parallel samples for both control and experimental groups.

### 2.5 Cornea Penetration Analysis

The cryoMN patch was thumb‐pressed into a 0.4% agarose gel or porcine cornea. Agarose gel was prepared by mixing agarose powder with ultrapure water under heat until it was completely dissolved. Porcine eyes were taken from 6‐ to 7‐month old pigs and collected from Primary Industries Pte Ltd (Singapore). After MN penetration, the agarose gel was imaged using a Zeiss LSM 800 confocal microscope. The appearance of the porcine cornea was recorded using a microlens‐equipped digital camera. MN‐treated porcine corneas were fixed with 4% paraformaldehyde for cryo‐sectioning and stained with hematoxylin and eosin (H&E) for histological analysis.

### 2.6 Ocular Delivery of Predatory Bacteria with cyroMNs in the Eye Infection Mouse Model

The antimicrobial efficacy of predatory bacteria as topical eye drops and incorporated in MN, along with an untreated control, was assessed in a mouse model of *E. coli* keratitis. We used 12 pathogen‐free 6‐ to 8‐week old male mice (wild‐type C57BL/6) per the SingHealth Institutional Animal Care and Use Committee (IACUC) guidelines (Protocol No. 2016/SHS/1204). For the animal experimentation, all the animals were handled as per the guidelines of Association for Research in Vision and Ophthalmology (ARVO). The mice were randomly distributed into three groups. Group I (the control) was topically treated with 0.9% NaCl, Group II was topically treated with *B. bacteriovorus* solution, and Group III was treated with *B. bacteriovorus‐*containing cryoMN patches. *E. coli* (ATCC 25922) was grown overnight in tryptic soy agar (TSA) plates at 37 °C. Isolated single bacterial colonies were picked and suspended in sterile saline at a concentration of 1–5 × 10^6^ CFU mL^−1^. Prior to the infection procedure, the eyes of the mice were examined by slit‐lamp photography and optical coherence tomography (OCT) to ensure the absence of corneal aberration (i.e., vascularization or other ocular defects). Mice were anesthetized by an intraperitoneal injection of xylazine (10 mg kg^−1^, Troy Laboratories, Smithfield, Australia) and ketamine (80 mg kg^−1^, Ketamine, Parnell Laboratories, Australia) under a dissecting microscope (Zeiss, Stemi‐2000 C). One drop of 1%–5% lidocaine hydrochloride was topically applied as anesthesia instilled before corneal wounding, and the corneal epithelium was then scratched using a sterile Beaver6400 Mini‐Blade to create a superficial wound without damaging the stroma. Next, the cornea was irrigated with sterile saline to remove any debris and residual topical anesthetic agents. Then, 15 µL of bacterial suspension containing 1–5 ×10^6^ CFU mL^−1^ of *E. coli* (ATCC 25922) was applied topically on the corneal surface. Six hours post infection (p.i.), mice were topically treated with 0.9% NaCl, *B. bacteriovorus* solution, or cryoMNs containing *B. bacteriovorus* three times per day for 3 days, with a 3 h interval between each application. Mouse eyes were then examined daily using a slit lamp and OCT.

### 2.7 Quantification of Viable Bacteria in the Mouse Cornea

On day 4, the mice were sacrificed, and their eyes were enucleated for bacterial quantification. The mouse corneas were dissected and individually homogenized in sterile PBS using a Pellet pestle cordless motor (Z359971, Sigma) with sterile plastic pestles. Homogenization was conducted by bead‐beating using sterile glass beads (2 mm). The resulting solution was diluted with sterile saline to give 10^–1^, 10^–2^, 10^–3^, 10^–4^, and 10^–5^ dilutions. An amount of 0.1 mL of each suspension was inoculated onto duplicate TSA plates. The plates were incubated at 37 °C for 24 h before the number of colonies was counted. The results are expressed as the log_10_ number of CFU/cornea.

## 3 Results

### 3.1 Fabrication and Characterization of cryoMNs

**Figure 1:**
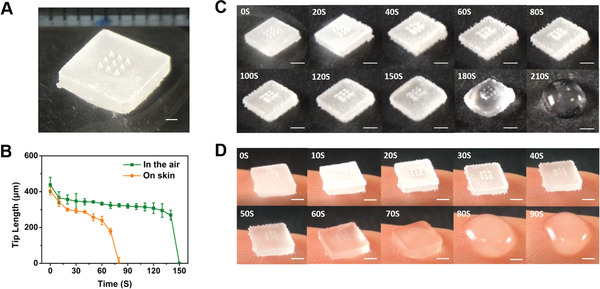
The morphology and melting behaviour of cryoMNs: A) Photographic image of intact cryoMNs (scale bar: 1 mm). B) Quantification of MN tip lengths after exposure to RT and body temperature; cryoMN melting behavior C) in the air; D) on human figure tips. Scale bar is 2 mm in both C and D.

The original stainless‐steel template (Figure S1, Supporting Information) had a 3 × 3 MN array with an interneedle spacing of 450 µm. Each MN tip had a height of 600 µm with a base width of 250 µm. This design was shown to fit the size of the mouse cornea in our previous study.^[^ ^8^ ^]^ Later, a PDMS negative mold was derived from this master template and used to prepare cryoMNs (Figure S2, Supporting Information). The cryoMN formulation was composed of 5% glycerol and *B. bacteriovorus* at concentrations ranging from 1 × 10^8^ to 1 × 10^9^ PFU mL^−1^. When the solution was loaded into the PDMS mold, low‐speed centrifugation was performed to load the bacteria into the tip cavities. Without centrifugation, it would require more than 60 min for 60% of the seeded bacteria to reach the tips through gravity (Figure S3, Supporting Information). After the freezing process, the cryoMNs were peeled off from the PDMS mold, and showed a morphology similar to that of the original master mold (**Figure** **1**A). We studied the stability of cryoMNs at both room temperature (RT, 24 °C) and body temperature (Figure 1B–D). The tip length of the cryoMNs was approximately 400–440 µm. When cryoMNs were removed from their cryopreservation environment (−80 °C) and placed under RT, frost appeared on the cryoMNs in 20 s (Figure 1C). After 150 s, the needle tips began to melt. When the cryoMNs were placed on a fingertip (37 °C, Figure 1D), the needle tips melted in 60 s. There was no pain or discomfort during the process of holding. The remaining needle tips were quantified and correlated with residence time to evaluate the survival window of the cryoMN. As shown in Figure 1B, cryoMNs maintained their morphology slightly longer at RT.

### 3.2 Optimization of cryoMN Formulation

**Figure 2:**
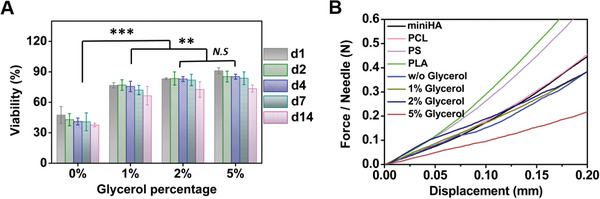
Optimization of cryoMN formulation: A) The viabilities of *B. bacteriovorus* cryopreserved in cryoMN formulations with different glycerol concentrations through the 14‐day storage, *N* = 5. B) The loading force‐displacement profiles of cryoMNs with different glycerol concentrations. *N* = 4. ^**^ *p* < 0.01, ^***^ *p* < 0.001, N.S means no significant difference.

We tuned the glycerol concentration between 0% and 5% and examined the viability of *B. bacteriovorus* inside cryoMNs in a 14‐day period post fabrication (**Figure** **2**A). In the absence of glycerol (0%) in the formulation, the viability of *B. bacteriovorus* reduced to less than 40% after 14 days. The addition of glycerol (1%–5%) significantly improved the viability to 80%–90%. When the glycerol concentration was more than 5%, the cryoMNs became very soft and easily melted during demolding (Figure S4, Supporting Information). CryoMN patches rapidly entered the liquid state after being removed from cryopreservation. We further examined the mechanical strength of cryoMNs with 0%–5% glycerol concentrations and compared them with those of polymeric MNs made from miniHA, PCL, PS, and PLA. As shown in Figure 2B, the cryoMNs with 0%, 1%, and 2% glycerol displayed similar loading force/displacement profiles to miniHA and PCL MNs in the compression test. They could withstand a load force of 0.3 to 0.4 N per needle without fracture. Higher glycerol concentration resulted in lower mechanical strength, but cryoMNs with 5% glycerol retained sufficient strength to penetrate the cornea (≈0.05 N per needle).^[^ ^8^ ^]^ Consequently, we employed cryoMNs with 5% glycerol for subsequent studies.

### 3.3 Cornea Penetration of cryoMNs

**Figure 3:**
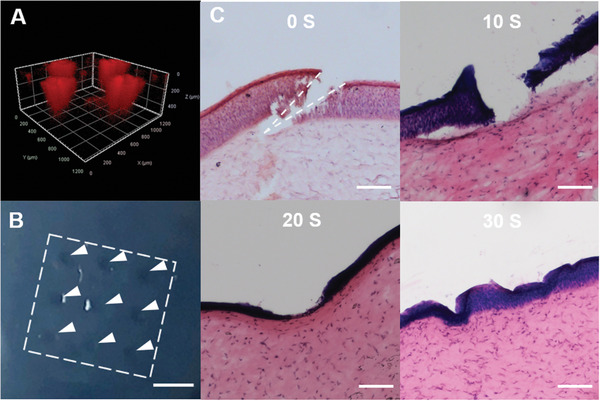
Penetration capability of cryoMNs into agarose gel and porcine cornea: A) Confocal image showing the penetration of cryoMNs and the delivery of bacteria in agarose gel. B) Image of porcine cornea with spots created by cryoMNs. The scale bar is 400 µm. C) H&E‐stained cross‐section images of porcine cornea after treatment with cryoMNs taken out from the freezer and left in the air for different times. The scale bar is 100 µm.

The penetration ability of the cryoMNs was first evaluated using agarose gel. To facilitate imaging, cryoMNs were loaded with *E. coli* stained with the red fluorophore FM 4–64FX. As shown in **Figure** **3**A and Figure S5 (Supporting Information), cryoMN easily pierced and delivered bacteria into the hydrogel. The penetration depth was less than 400 µm, which was slightly shorter than the actual length of the needles (400–450 µm).

Next, cryoMNs were tested on ex vivo porcine eyes by thumb pressing into the corneal region (Figure S6, Supporting Information). As shown in Figure 3B, obvious MN patterns were observed in the eye. Tissue histology showed that cryoMN tips broke through the corneal layer (762–898 µm thickness,^[^ ^14^ ^]^ Figure 3C) and penetrated into the corneal stromal layer (≈150 µm deep), which is about one‐third of the MN height. As cryoMNs underwent dissolution at RT after removal from the storage freezer (Figure 1B), we examined their corneal penetration capabilities at different time points after retrieval. As shown in Figure 3C, penetration of the corneal layer was still possible within 10 s after retrieval. However, the penetration ability was reduced significantly when the cryoMN was left at RT for a longer duration. These results suggest that cryoMNs can be used for bacterial delivery only if the operation time between the removal from storage and skin insertion is less than 10 s at RT. It can be expected that a longer operation time would be possible if the operating environment temperature was lower.

### 3.4 In Vitro Predation Test to Gram‐Negative Bacteria

**Figure 4:**
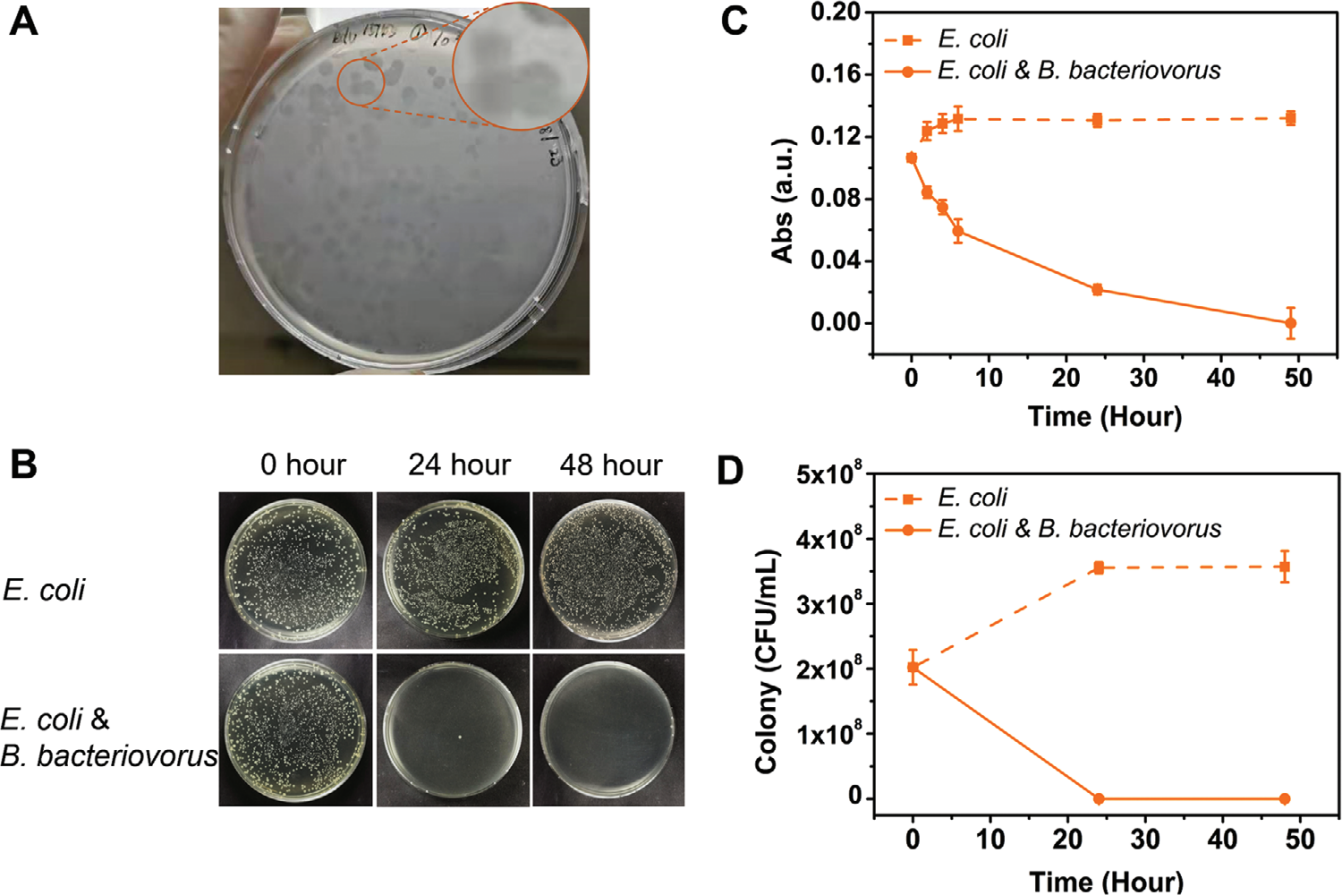
In vitro predation efficiency of *B. bacteriovorus* predatory bacteria delivered using cryoMNs against *E. coli* (ATCC 25922). A) *B. bacteriovorus* can “eat” *E. coli* and form clear spots on plates. B) Optical images of colony‐forming unit (CFU) plating (dilution factor of 10^–4^). Time‐dependent changes of *E. coli* concentrations with or without cryoMN treatment measured (C) by optical density at 600 nm and (D) by CFU plating.

The predatory capability of free *B. bacteriovorus* against gram‐negative bacteria was first confirmed with *E. coli* (ATCC 25922) (**Figure** **4**A and S7, Supporting Information). As shown in Figure S7A (Supporting Information), the absorbance value of *E. coli* incubated with *B. bacteriovorus* did not increase during the 48h period, while the absorbance value tripled for the untreated *E. coli* group. This observation was corroborated by colony counting results (Figure S7B, Supporting Information). *E. coli* concentration in the predated group decreased dramatically from 4.3 × 10^8^ to 6500 CFU mL^−1^ within 24 h of incubation, indicating a 4.8 log_10_ reduction compared to the untreated group. The log reduction value remained similar even after 48 h, suggesting no *E. coli* regrowth.

Next, we confirmed the predation efficiency of *B. bacteriovorus* following encapsulation and delivery with cryoMN patches against *E. coli* (ATCC 25922), *P. aeruginosa* (PAO1‐GFP), *A. baumannii*, and *K. pneumoniae*. Studies have suggested that these pathogens are the underlying causes of eye or skin infections.^[^ ^15^ ^]^ A predation experiment was conducted by co‐culturing the pathogens with predators recovered from the cryoMN. Except for PAO1‐GFP, the remaining pathogens were susceptible to predation by *B. bacteriovorus*. We quantified the eradication of these pathogenic bacteria by *B. bacteriovorus* through both optical density measurement and bacterial enumeration after 48 h of incubation. Figure 4B–D shows the concentration change of *E. coli* without or with *B. bacteriovorus* cryoMN treatment. The optical density of *E. coli* steadily increased in the untreated *E. coli* only group, while a dramatic decrease was observed in the co‐culture predated group (Figure 4C). Colony changes showed a similar trend to that of absorbance reading, with a 4 log_10_ reduction observed in the co‐culture predated group (Figure 4B–D). Representative photographs of agar plates for the control and co‐culture groups after 48 h are shown for better clarity. As shown in Figure 4B, bacterial colonies were completely cleared in the co‐culture group, whereas numerous colonies were apparent in the untreated control group.

**Figure 5:**
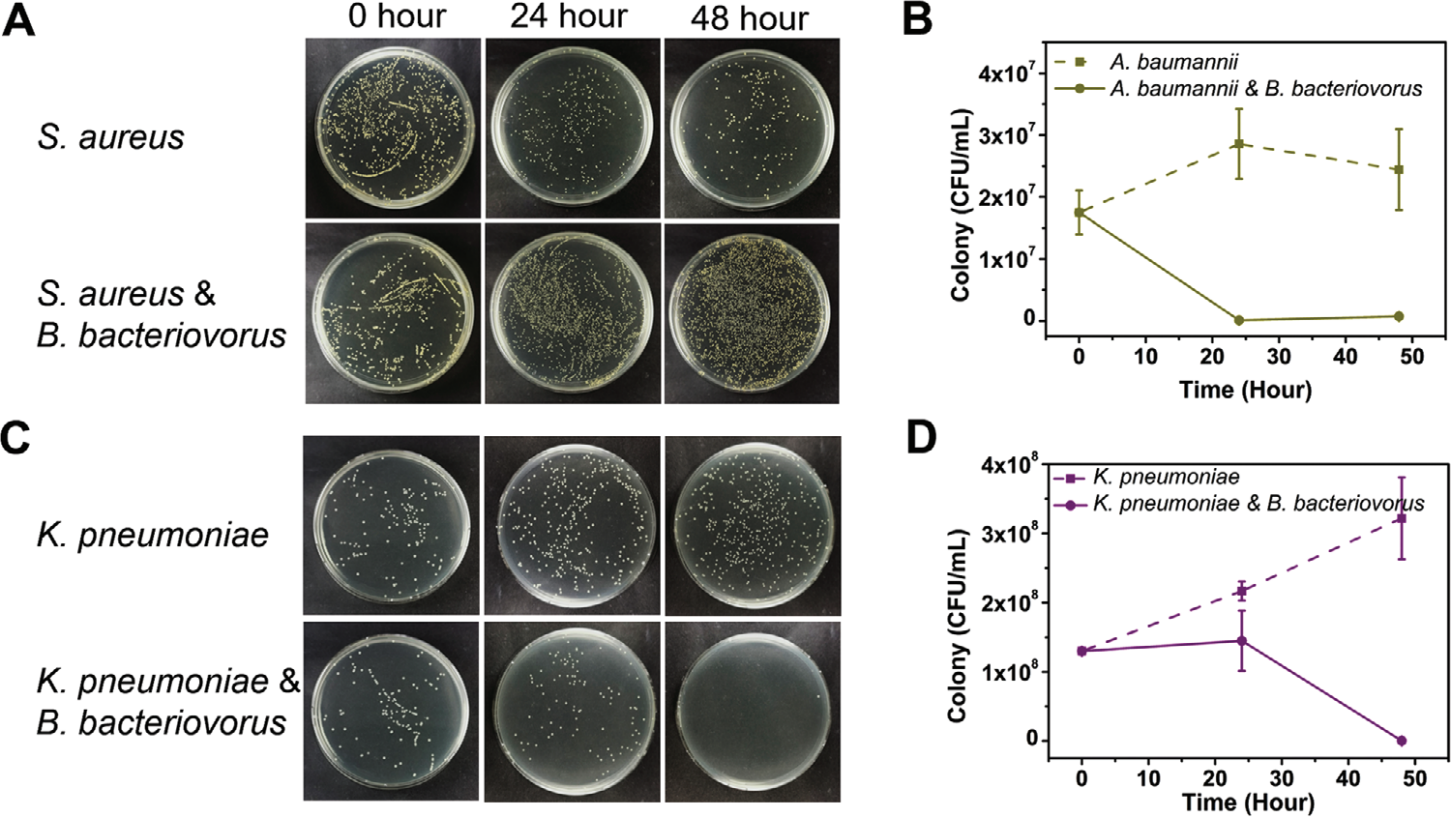
In vitro predation effects of *B. bacteriovorus* predatory bacteria delivered using cryoMNs against *A. baumannii* and *K. pneumoniae*. Optical images of agar plates (A) and quantification (B) showing the change of *A. baumannii* concentration without or with *B. bacteriovorus* cryoMNs (dilution factor of 10^–4^). Optical images of agar plates (C) and quantification (D) showing the change of *K. pneumoniae* concentration without or with *B. bacteriovorus* cryoMNs (dilution factor of 10^–5^).

We further examined the predation of *A. baumannii* and *K. pneumoniae* by *B. bacteriovorus*, which resulted in ≈ 3 log_10_ reductions for both strains after 48 h (**Figure** **5**). Looking at their concentration profiles, we noticed considerable differences between the two strains. While the number of *A. baumannii* bacteria drastically decreased during the first 24 h (Figure 5A,B), the *K. pneumoniae* group only showed a significant decrease on the second day (from 24 to 48 h; Figure 5C,D). The differences in outer membrane structure and natural adaptation of the two pathogenic microbes may contribute to the lower lethality rate against *K. pneumoniae*. Compared to the significant predation efficiency for the above three bacteria, no predation was observed for PAO1‐GFP bacteria (Figure S8, Supporting Information). Colony counting revealed that both untreated PAO1‐GFP and predated PAO1‐GFP groups significantly increased on the second day, following a minor decrease in the first 24 h. It was previously reported that *B. bacteriovorus* may not be able to consume all gram‐negative strains. For example, certain *B. bacteriovorus* can only prey on selective *P. aeruginosa* strains, such as Pa16. Shanks et al. demonstrated that only 70% of the tested *P. aeruginosa* strains were predated by the *B. bacteriovorus* 109 J strain. Meanwhile, the *B. bacteriovorus* HD100 strain was able to prey on all of the tested *P. aeruginosa* strains.^[^ ^4^ ^]^ Therefore, careful selection of the predatory strain is also crucial to ensure effective predation of pathogens.^[^ ^4^, ^15^ ^]^

### 3.5 Ocular Delivery of *B. bacteriovorus* with cryoMNs for Eye Infection

**Figure 6:**
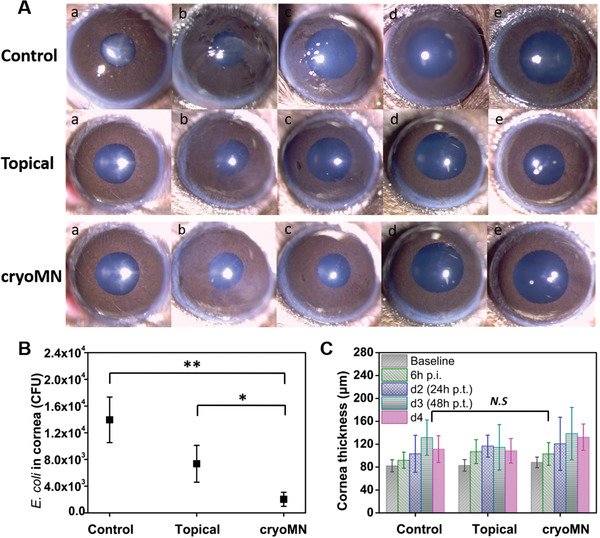
Ocular delivery of *B. bacteriovorus* with cryoMNs for eye infection: A) Cornea images taken by slit‐lamp photography (a. Baseline; b. 6 h p.i. after inoculation (prior treatment); c. Day 2 (24 h post treatment (p.t.)); d. Day 3; e. Day 4. B) Final *E. coli* concentration inside mouse corneas. *N* = 4. C) Cornea thickness before and after treatment every day. *N* = 4. ^*^ *p* < 0.1, ^**^ *p* < 0.01, N.S means no significant difference.

The efficacy of cryoMN therapy was evaluated in a mouse model of infectious keratitis. At 6 h post inoculation of *E. coli* (ATCC 25922) of scarified cornea, clear signs of infection in terms of corneal haze were observed by slit‐lamp bioimaging (**Figure** **6**A). The images indicated significant opacity around the pupillary area. To this end, treatment was conducted twice with a 3 h gap on the first day, and three times (3 h gap) on the second and third days. The eyes were examined by slit lamp and OCT prior to each treatment, and the mice were sacrificed on day 4 to isolate and quantify the bacteria in the cornea. With increasing time, the sham controls progressed toward enhanced corneal haze and stromal infiltrates, whereas corneas that received treatment with predatory bacteria showed clear or slight opacity around the pupil area. In support of these observations, bacterial bioburden in the cornea determined by bacterial enumeration indicated a marked decrease in bacterial titer for both topically applied and MN application. These results indicate the predation efficiency of *B. bacteriovorus* in vivo (Figure 6B). Notably, *E. coli* concentration was lowest in the cryoMN group, 5.8‐fold lower relative to the control group, and 2.6‐fold lower than that in the topical group. Corneal thickness was evaluated daily before each treatment. Interestingly, the topical group showed comparable results to the control and cryoMN groups (Figure 6C).

## 4 Discussion

The excessive and inappropriate use of antibiotics has resulted in the evolution of multi‐drug and pan‐drug resistant bacteria and undermined the effectiveness of antibiotics, thus limiting treatment options. The waning antibiotic pipeline and unfavorable economic incentives exacerbate the current antibiotic crisis, thus warranting the need for new approaches to infection control. This study investigated the use of “living antibiotics” and predatory bacteria as a new class of armamentarium against pathogenic bacteria in an eye infection model.^[^ ^17^ ^]^Additionally, to facilitate delivery, we upgraded the cryoMN platform to maximize the preservation of predatory bacteria activity during preparation, storage, and deployment.

This formulation consisted of two major components, namely the cryoprotectant medium and the live therapeutics – predators (e.g., *B. bacteriovorus*). We first determined that 5% glycerol was an optimal medium, conferring sufficient mechanical strength and integrity while retaining the viability of *B. bacteriovorus* at greater than 80% (Figure 2). *B. bacteriovorus* was chosen as the model predator here because of its unique ability to predate gram‐negative bacteria.^[^ ^5^, ^18^ ^]^ It was previously shown to be capable of feeding on multi‐drug resistant bacteria (e.g., colistin‐resistant *K. pneumoniae*).^[^ ^16^ ^]^ A recent study revealed that the higher the dosage of *B. bacteriovorus*, the greater the predation effect in the biolysis of sewage sludge.^[^ ^19^ ^]^ Moreover, no adverse effects or loss of host viability were observed in mice after repeated administration of high doses of predatory bacteria (4 ×  10^9^ PFU per mouse, 3 doses), and predators were quickly and efficiently cleared from the mice within 10 days post‐injection.^[^ ^5^, ^13^, ^20^ ^]^

The derived cryoMN device was able to disrupt the barrier function of the porcine cornea in ex vivo testing (Figure 3). Dissolvable MNs have been used to deliver probiotics in powder form.^[^ ^21^ ^]^ However, the maximum loading efficiency was approximately 1.5 × 10^4^ CFU per patch with 81 needle tips, which is significantly lower than that in our design. Here, 8 × 10^6^ PFU predator cells were loaded into one patch with only nine tips. Moreover, in its powder form, the functionality of *B. bacteriovorus* is impeded, necessitating a liquid formulation to trigger the release of predatory bacteria.^[^ ^22^ ^]^ In contrast, the activation of *B. bacteriovorus* after the deployment of the cryoMN patch is rapid (within 80 s) and they are then able to prey on the bacteria within 18 to 24 h, minimizing the time lag and significantly saving on initiation time. We examined the predation efficiency of *B. bacteriovorus* both in the free form and in the cryoMN formulation (Figure 4, Figure 5, Figure S7, Supporting Information). Both CFU plating and the optical density method confirmed that the predation capability of *B. bacteriovorus* was fully retained in the cryoMN formulation. Additional testing with *A. baumannii* and *K. pneumoniae* revealed the potential of cryoMNs carrying *B. bacteriovorus* to treat keratitis and endophthalmitis, respectively. However, *B. bacteriovorus* did not effectively prey on PAO1‐GFP strains (Figure S8, Supporting Information), indicating the need for pre‐identification of the pathogen strains before treatment. Finally, we used a mouse eye infection model to demonstrate the clinical potential of this technology (Figure 6). Compared with the topical delivery of *B. bacteriovorus*, cryoMN‐aided delivery significantly improved the effectiveness of the treatment.

Despite these achievements, several challenges exist for cryoMN formulations. First, the current formulation was preserved and transported at−80 °C. A formulation that is suitable for preservation and transport at a higher temperature would save costs and offer greater convenience. Second, cryoMNs can potentially induce discomfort to the eye cornea at a very low temperature. Formulations that function at higher temperatures would mitigate this issue also. Finally, the speed and force for MN application to the eye must be precisely controlled to avoid damage to the delicate cornea. An automated applicator system is beneficial for solving this issue.^[^ ^23^ ^]^

## 5 Conclusion

This study describes an MN system for the ocular delivery of living bacteria. The device was characterized and tested in both in vitro and in vivo settings. In cell experiments, the predatory bacteria (*B. bacteriovorus*) delivered with this device successfully suppressed the proliferation of gram‐negative *E. coli, A. baumannii*, and *K. pneumoniae*. In the mouse eye infection model, *B. bacteriovorus* delivered via cryoMN significantly reduced the *E. coli* concentration in the cornea relative to the control or topical treatment. The concept presented here is versatile and can be expanded to other predators to prey on specific target pathogens. Future studies will aim to achieve formulations with higher working temperatures and a coupling applicator system to ensure safe and controlled deployment.

## Conflict of Interest

The authors declare no conflict of interest.

## Supporting information

Supporting Information

## Data Availability

Research data are not shared.
