# Supplementary material for: Ocular Delivery of Predatory Bacteria with Cryomicroneedles Against Eye Infection
Source: Adv Sci (Weinh). 2021 Sep 8;8(21):2102327. doi: 10.1002/advs.202102327 (PMC8564459; doi:10.1002/advs.202102327)
Supplement: Supplementary file 1 — Supporting Information [file ADVS-8-2102327-s001.pdf]

© 2021 Wiley-VCH GmbH

ADVANCED SCIENCE
Open Access

Supporting Information

for *Adv. Sci.,* DOI: 10.1002/advs.202102327

Ocular delivery of predatory bacteria with cryo-microneedles against eye infection

Mingyue Cui[1,2], Mengjia Zheng[1,2], Christian Wiraja[2], Sharon Wan Ting Chew[2], Arti Mishra[3], Venkatesh Mayandi[3], Rajamani Lakshminarayanan[3,4,5], Chenjie Xu[1*]*

Supporting information

Ocular delivery of predatory bacteria with cryo-microneedles against eye infection

Mingyue Cui[1,2], Mengjia Zheng[1,2], Christian Wiraja[2], Sharon Wan Ting Chew[2], Arti Mishra[3], Venkatesh Mayandi[3], Rajamani Lakshminarayanan[3,4,5], Chenjie Xu[1*]*

1 Department of Biomedical Engineering, City University of Hong Kong, 83 Tat Chee Avenue, Kowloon, Hong Kong SAR, China

2 School of Chemical and Biomedical Engineering, Nanyang Technological University, 62 Nanyang Drive, Singapore 637459

3 Ocular Infections & Anti-Microbials Research Group, Singapore Eye Research Institute, The Academia, 20 College Road, Discovery Tower, Singapore 169856

4 Ophthalmology and Visual Sciences Academic Clinical Program, Duke-NUS Graduate Medical School, 8 College Road, Singapore 169857

5 Department of Pharmacy, National University of Singapore, 18 Science Drive, Singapore 117543

* Correspondence to chenjie.xu@cityu.edu.hk; lakshminarayanan.rajamani@seri.com.sg

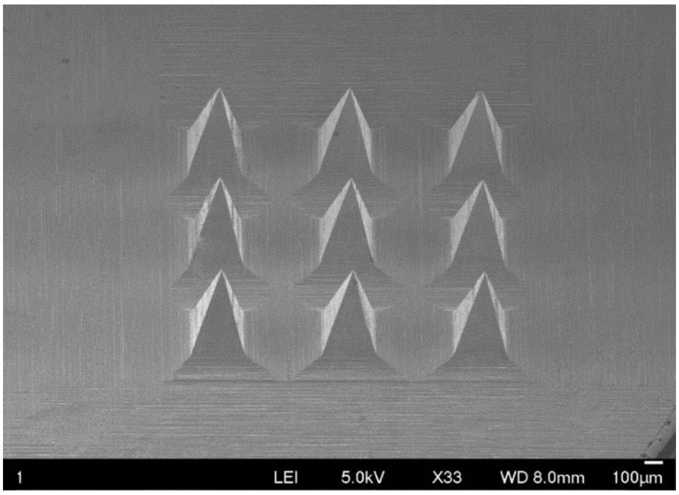

Figure S1. Scanning Electron Microscope image of stainless-steel microneedle (MN) mother template (scale bar is 100 μm).

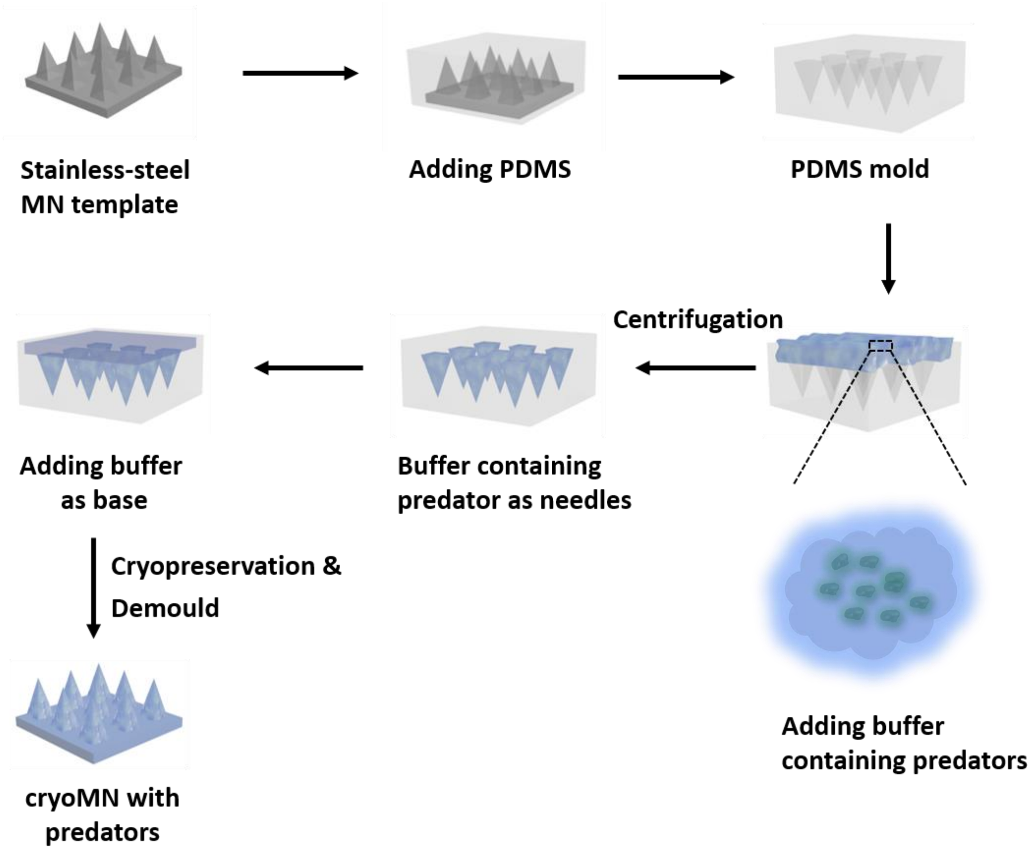

Figure S2. Schematic illustration of the cryo-microneedle (cryoMN) fabrication process.

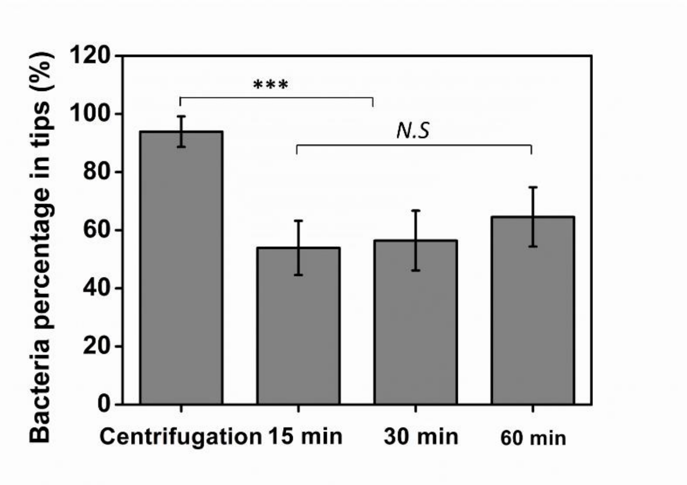

Figure S3. Percentage of bacterial inside needle tips by either centrifugation or natural sediment.

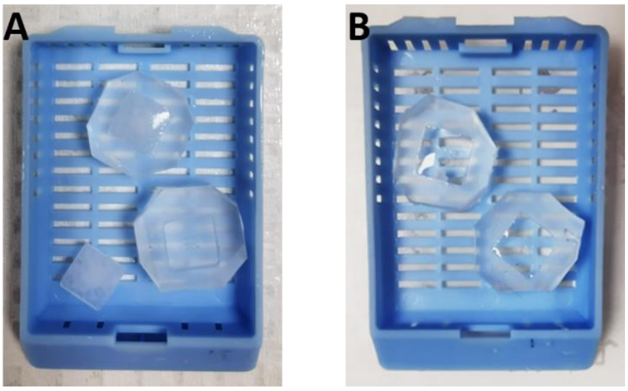

Figure S4. cryoMN patches made of cryoprotectant medium containing (**A**) 5% or (**B**) 10% glycerol.

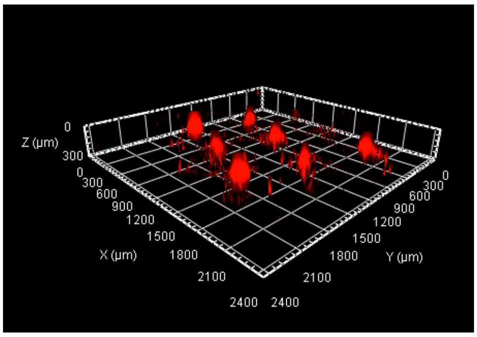

Figure S5. Confocal image showing the penetration of cryoMNs and the delivery of bacteria in agarose gel.

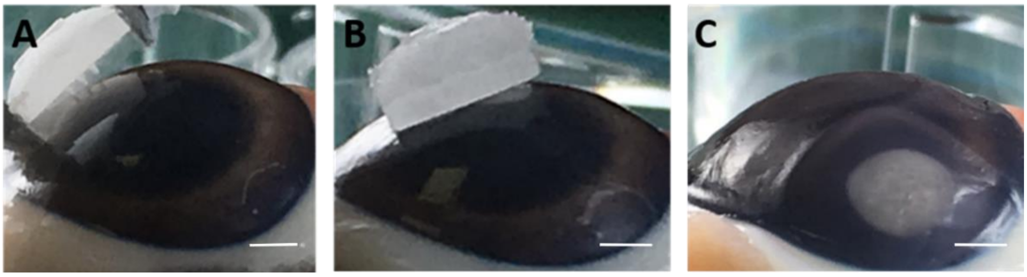

Figure S6. Cornea penetration of cryoMNs. A cryoMN patch containing 3 × 3 MNs was thumb pressed on the central region of porcine eye. Bright-field images of the porcine eye before (**A**) and after (**B**) cryo-MN insertion. (**C**) A close view of the MN patterns left on the eye. Scale bar is 2 mm.

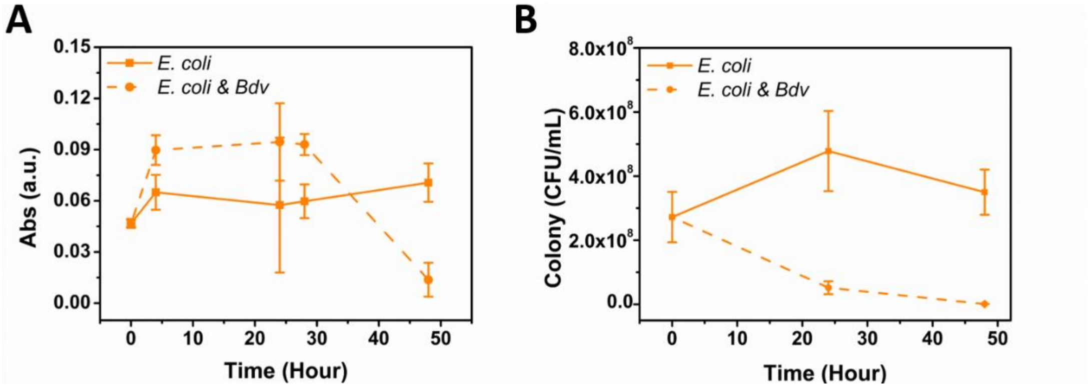

Figure S7. *In vitro* predation ability of predatory bacteria. (**A**) Time-dependent optical density changes after incubation with prey cells measured by BioTek plate reader. (**B**) Time-dependent changes of bacterial concentrations after incubation with prey cells measured by CFU plating.

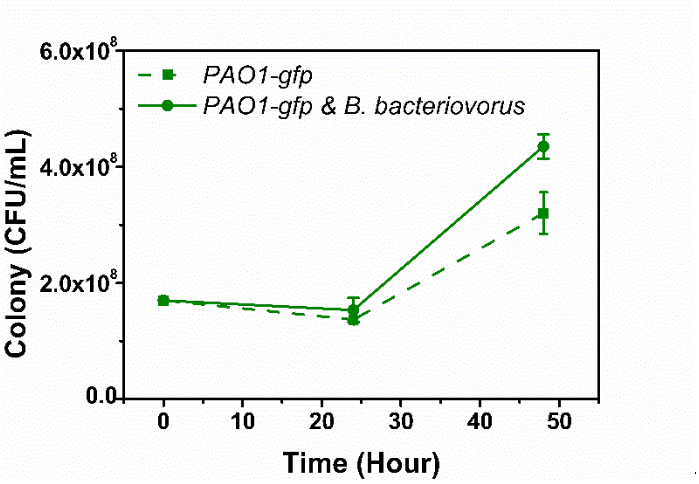

Figure S8. *In vitro* predation of *B. bacteriovorus* delivered by cryoMNs against PAO1-gfp.
